# Beating the amorphous limit in thermal conductivity by superlattices design

**DOI:** 10.1038/srep14116

**Published:** 2015-09-16

**Authors:** Hideyuki Mizuno, Stefano Mossa, Jean-Louis Barrat

**Affiliations:** 1 Univ. Grenoble Alpes, LIPHY, F-38000 Grenoble, France; 2 CNRS, LIPHY, F-38000 Grenoble, France; 3 Univ. Grenoble Alpes, INAC-SPRAM, F-38000 Grenoble, France; 4 CNRS, INAC-SPRAM, F-38000 Grenoble, France; 5 CEA, INAC-SPRAM, F-38000 Grenoble, France; 6 Institut Laue-Langevin - 6 rue Jules Horowitz, BP 156, 38042 Grenoble, France

## Abstract

The value measured in the amorphous structure with the same chemical composition is often considered as a lower bound for the thermal conductivity of any material: the heat carriers are strongly scattered by disorder, and their lifetimes reach the minimum time scale of thermal vibrations. An appropriate design at the nano-scale, however, may allow one to reduce the thermal conductivity even below the amorphous limit. In the present contribution, using molecular-dynamics simulation and the Green-Kubo formulation, we study systematically the thermal conductivity of layered phononic materials (superlattices), by tuning different parameters that can characterize such structures. We have discovered that the key to reach a lower-than-amorphous thermal conductivity is to block almost completely the propagation of the heat carriers, the superlattice phonons. We demonstrate that a large mass difference in the two intercalated layers, or weakened interactions across the interface between layers result in materials with very low thermal conductivity, below the values of the corresponding amorphous counterparts.

Materials with low thermal conductivity, *κ*, are employed in many modern technologies, such as thermal management in electronic devices or thermoelectric energy conversion^1^,^2^,^3^. In general, low values of *κ* are observed in disordered solids^4^, including topologically disordered systems and crystalline solids with size or mass disorder^5^,^6^,^7^,^8^. We refer to the former as glasses, to the latter as disordered alloys. While in glasses disorder originates both from molecules size or mass heterogeneity and the topologically amorphous structure, in disordered alloys molecules characterized by heterogeneous attributes are located at ordered lattice sites. The low *κ* in disordered solids can be rationalized by considering the phenomenological kinetic theory expression^9^ *κ* = (1/3)*Cv*^2^*τ*, which relates average velocity, *v*, and lifetime, *τ*, (and therefore the mean free path 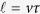) of phonons to *κ* (*C* is the specific heat per unit volume). In good crystals, phonons lifetime is primarily controlled by anharmonic interactions. In contrast, in disordered solids, the disorder (or the elastic heterogeneity^10^) reduces *τ* (or 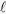) and, as a result, *κ*.

In early experimental investigations^5^,^6^, Cahill *et al.* have studied the disordered alloys, e.g., (KBr)_1−*x*_(KCN)_*x*_, (NaCl)_1−*x*_(NaCN)_*x*_, and shown that *κ* can be reduced to the glass value by controlling the relative composition *x*. In our work^8^ we in turn demonstrated that, in size-disordered crystal (alloy), *κ* progressively decreases with increasing size mismatch, eventually converging to the corresponding glass value. When this limit is reached, *τ* is comparable to the time scale of thermal vibrations (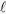 to the particle size), i.e., to the minimum time (length) scale^8^. Heat propagation can therefore be described as a random walk of vibrational energies^5^,^6^, or in terms of non-propagating delocalized modes, the diffusons^7^. For this reason, the value in the glass is generally considered as a lower bound for *κ* of materials with homogeneous chemical composition^5^,^6^.

A crucial issue^4^ is whether thermal conductivity can be lowered below the glass limit through nanoscale phononic design^3^,^11^. This possibility would allow to devise (meta-)materials which are excellent thermal insulators while preserving good electronic properties, as needed in many applications^1^,^2^,^3^. The most popular design to reach this goal is that of a lamellar superlattice^12^,^13^,^14^,^15^, often composed of two chemically different intercalated layers, e.g., Si-Ge^12^,^13^ or GaAs-AlAs^14^,^15^ (see also Fig. 1). In a superlattice, the thermal conductivity tensor is anisotropic, with the cross-plane component, *κ*_CP_, usually lower than the in-plane value, *κ*_IP_^16^,^17^.

In recent experiments^18^,^19^,^20^, ultra-low values of *κ*_CP_, suggested to be smaller than the amorphous limit, were measured. In particular, Costescu *et al.*^18^ demonstrated that the presence of a high-density of interfaces decreases *κ*_CP_ of W-Al_2_O_3_ nanolaminates, below that of the amorphous Al_2_O_3_. An experiment by Chiritescu *et al.*^19^ achieved ultra-low thermal conductivity in layered WSe_2_ crystals, by disordering the crystalline WSe_2_ sheets. Finally, Pernot *et al.*^20^ also observed very low values of *κ*_CP_, below that of amorphous Si, in Ge nanodots multi-layers separated by Si crystals.

Although the above experimental works have demonstrated very low values of *κ* in superlattice systems, we note that these values have not been systematically compared to those assumed in the glasses with *exactly* the same chemical composition. Since different chemical species are expected to produce different effects on *κ*, it is therefore still not completely clear whether superlattice structuration alone can lower *κ* below the amorphous limit. Note, for instance, that very recent numerical work^21^, has shown that a superlattice composed by layers with randomized thicknesses can indeed show a *κ* below the value pertaining to the disordered-alloy with the same composition. This limit, however, is generally higher than that in the corresponding glass^5^,^6^,^7^,^8^, which should therefore be considered the true amorphous limit to be beaten.

In addition, a general framework to rationalize in a coherent single picture the previous observations of very low *κ* is, to the best of our knowledge, still lacking.

In this work, we address these two issues. Building on the comparison of the superlattice with the corresponding amorphous structure, we clarify the mechanisms allowing for ultra-low thermal conductivity in the former. We have studied by computer simulation a numerical model that allows one to exactly compare ordered and disordered systems with identical chemical composition and access detailed information on the entire normal modes spectrum, providing, as a consequence, a complete understanding of the heat transfer process. As the lifetime of heat carriers is already minimum in glasses^8^, we demonstrate that the key to even lower thermal conductivities is to suppress their propagation across the interfaces between the constituent layers.

More in details, we have focused on three distinct design principles for superlattices, mimicking similar configurations actually employed in experiments. These are based on the face-centered-cubic (FCC) lattice structure, and are composed of: (*S1*) two intercalated crystalline layers formed by point particles with different masses; (*S2*) ordered crystalline layers intercalated to mass-disordered alloy layers; and (*S3*) identical crystalline layers with modified (weakened) interactions across the interfaces (see the **Methods** section and Table 1). We show that a large mass difference between layers (*S1*) and weakened interactions between layers (*S3*) efficiently obstruct the propagation of phonons, resulting in a very large reduction of the superlattice thermal conductivity, even below the values pertaining to the glass phases with identical composition. Based on our results, we conclude with a discussion of the optimal strategy to follow towards *very* low thermal conductivity materials.

In Fig. 1 we show a schematic illustration of a superlattice composed by two intercalated layers, *A* and *B*, both of thickness *W*/2. The competition between two length scales, the repetition period of the superlattice, *W*, and the mean free path of the superlattice phonons, 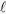, determines the coherent or incoherent character of phonon transport, as described in^22^,^23^,^24^ and demonstrated by numerical simulations^25^,^26^,^27^ and recent experiments^28^.

For 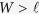, the *incoherent* phonon transport is independent in the different layers, and phonons can be effectively treated as particles. In this case, the Boltzmann transport equation applies^29^,^30^, and the particle-like phonons are scattered within the layers (internal resistance) and at the interfaces (interfacial resistance or Kapitza resistance^31^,^32^,^33^). The thermal conductivity in the cross-plane direction can be written as^22^


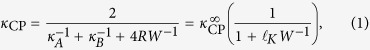


where


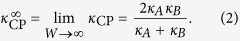


Here, *κ*_*A*_ and *κ*_*B*_ are the thermal conductivities of materials *A* and *B*, and 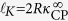 is the Kapitza length^34^. *R* is the interfacial resistance, which exists even at a perfect interface and depends on the nature of the contacting materials (e.g., crystal-crystal, crystal-glass)^31^,^32^. For 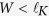 (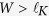), the interfacial resistance is relatively large (small) compared to the internal resistance. Both *κ*_CP_ and *κ*_IP_ (the in-plane thermal conductivity) increase with *W*, due to the decrease of the interfacial resistance density^29^,^30^. In the diffuse limit *W* → ∞, where the interfacial resistance can be neglected, *κ*_CP_ and *κ*_IP_ have the upper bounds 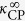 and 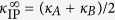, respectively.

When 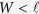, phonon transport is *coherent*^22^,^23^,^24^,^25^,^26^,^27^,^28^, and the wave nature of phonons cannot be neglected. In this regime, *κ*_CP_ decreases with increasing *W*, in contrast with the incoherent case. The reduction of *κ*_CP_ is explained with the emergence of a band gap at the Brillouin zone boundary, due to band-folding^35^: increasing *W* augments the frequency gap in the dispersion relation. This, in turn, decreases the average group velocity *v* of phonons, finally reducing *κ*_CP_. Mini-umklapp processes^36^, occurring at the mini-Brillouin zone, also contribute to the reduction of *κ*_CP_. At the crossover length 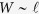, between the incoherent and the coherent transport regimes, *κ*_CP_ assumes a minimum value when plotted against *W*^22^,^23^,^24^,^25^,^26^,^27^,^28^. We have encountered this situation in the case of superlattice *S1*, as we will see below.

Details of the structure of the interface between layers are also known to significantly affect phonon transport^37^,^38^,^39^,^40^,^41^,^42^,^43^,^44^,^45^,^46^. It has been reported that interfacial roughness^37^,^38^,^39^ or mixing^40^,^41^ reduce both *κ*_CP_ and *κ*_IP_, and can even suppress the coherent nature of phonons, with *κ*_CP(IP)_ increasing monotonously at any *W*. The interface topology is also an important factor to determine the phonon transport^42^,^43^. While we will not address precisely this situation in detail here, the superlattice *S2* of our study bears some similarities with it.

Finally, the stiffness of interfacial bondings, which can be controlled by applying pressure^44^,^45^ or tuning chemical bonding^46^, has significant effects on heat transport features, which will be demonstrated by the study of the *S3* superlattice.

## Results

In Table 1, we present the details of the three superlattice systems studied in this work, with values of the important quantities: *κ*_*A*_ and *κ*_*B*_ are the thermal conductivities of layers *A* and *B*, respectively; 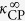 and 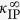 are the cross- and in-plane diffuse limits of *κ*_CP_ and *κ*_IP_; *R* is the interfacial resistance, 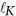 the Kapitza length; *κ*_glass_ and *κ*_alloy_ are the thermal conductivities of the glass and disordered alloy with exactly the same composition as the indicated superlattice.

The number density of all systems was fixed at 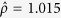, with a corresponding crystal lattice constant *a* = 1.58. In order to minimize anharmonic couplings and focus primarily on the contribution arising from the details of the nano-structuration, we considered a low temperature value *T* = 10^−2^.

**Figure 1:**
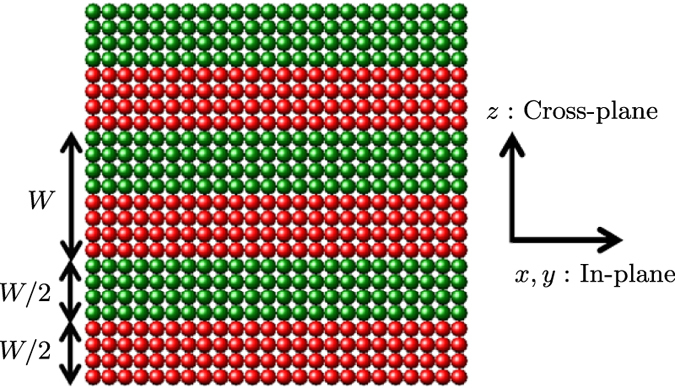
Schematic illustration of the considered superlattice structures. The superlattice is composed of two FCC-lattice layers, *A* (red) and *B* (green). The two layers have identical thickness *W*/2, where *W* is the replication period. Here, we measure *W* as the number of monolayers of the lattice structure, e.g., *W* = 8 in the displayed case. The distance between adjacent monolayers is *a*/2 for the perfect FCC structure, where *a* is the lattice constant.

Thermal conductivities have been estimated by the Green-Kubo formulation^47^,^48^. We have calculated both components of the superlattices thermal conductivities, *κ*_CP_ and *κ*_IP_, by varying the pattern repetition period *W*. Note that *W* indicates the number of monolayers of the lattice structure, where the distance between adjacent monolayers is *a*/2 for the perfect FCC structure (see also Fig. 1). In the following, we systematically compare the value of *κ*_CP_ to 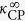, *κ*_glass_, *κ*_alloy_, to evaluate the efficiency of the superlattice structures in minimizing heat transfer in the direction of the patterning. The in-plane behaviour has been similarly quantified by comparing *κ*_IP_ to 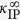. In addition, we have also characterized the vibrational states by using a standard normal-modes analysis^8^,^49^.

All important information about the system models and methods used for the simulation production runs and analysis are given in the **Methods** section. Additional details about specific points are included in the Supplementary Materials.

### S1. Superlattice composed of two intercalated crystalline layers with different masses

**Figure 2:**
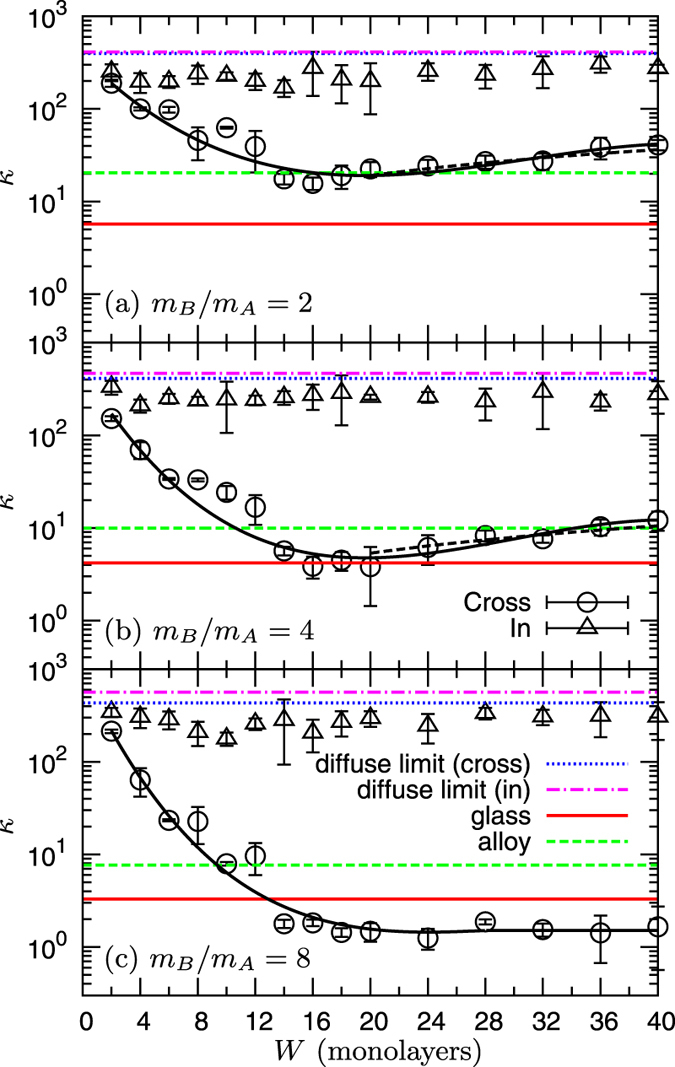
Thermal conductivity in superlattice *S1* composed of two intercalated crystalline layers with different masses. The cross-plane, *κ*_CP_, and in-plane, *κ*_IP_, components of thermal conductivity are plotted as functions of the repetition period *W*. The ratio *m*_*B*_/*m*_*A*_ of the masses in layers *A* and *B* is 2 in panel (**a**), 4 in (**b**), and 8 in (**c**). The values 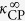 and 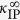 of the diffuse limits (*W* → ∞), as well as those in the glass and the disordered alloy with the same constituent species are indicated by the horizontal lines. In panels (**a**) and (**b**) we also show (dashed black lines), the prediction of Eq. (1) for *κ*_CP_ in the incoherent regime, *W* > 20, with the values of *R* and 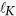 included in Table 1. The solid curve interpolating the *κ*_CP_ data points in the entire *W*-range is a guide for eye. The calculation of the displayed error bars is detailed in the **Methods** section.

In Fig. 2 we show the thermal conductivities, *κ*_CP_ and *κ*_IP_ (symbols), as functions of the replication period, *W*, for the layers mass ratios *m*_*B*_/*m*_*A*_ = 2, 4, and 8. Note that we have chosen to fix the average mass, 〈*m*〉 = (*m*_*A*_ + *m*_*B*_)/2 = 1, rather than fixing a reference value *m*_*A*_ = 1 and varying *m*_*B*_. This latter protocol has indeed an obvious drawback: the average mass would increase when considering different values of the ratio *m*_*B*_/*m*_*A*_, implying a trivial effect on the thermal conductivity which scales as 〈*m*〉^−1/2^. This would therefore hinder the possibility to isolate the contribution to lowering the thermal conductivity which originates from the mass mismatch alone.

The values of the diffuse limits 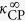 and 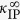 as well as those of the glass and the disordered alloy constituted by the same species (see Table 1) are also shown as lines in Fig. 2. As expected, the relation 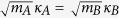 holds for the pure materials. In the studied *W*-range, *W* = 2 to 40 (monolayers), the in-plane value *κ*_IP_ shows a very weak dependence on *W*, as was observed for superlattices with perfect interfaces in Refs 26,39. The value of *κ*_IP_ is close to, although lower than, 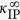, indicating that slight in-plane phonon scattering at the interface is still active.

More interestingly, as *W* increases, the cross-plane value *κ*_CP_ decreases steeply, reaches a minimum value at 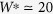, and next increases mildly at larger *W*. This *W*-dependence is consistent with previous predictions^22^,^23^,^24^,^25^,^26^,^27^,^28^, and corresponds to the crossover at W^*^ from coherent to incoherent phonon transport. In the incoherent regime, *W* > 20, from Eq. (1) and the data of *κ*_CP_ (dashed line in Fig. 2) we can extract the values of the interfacial resistance, *R*, and the Kapitza length, 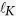, which are presented in Table 1.

Note that for *m*_*B*_/*m*_*A*_ = 8 (Fig. 2(c)), we do not observe a clear thermal conductivity minimum. More precisely, even at the largest value *W* = 40, *κ*_CP_ is still orders of magnitude lower than 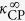, indicating that the interfacial resistance *R* results in a strong reduction of *κ*_CP_ in this range of *W*. Equivalently, the Kapitza length 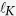 is significantly larger than the maximum period *W* = 40. Also, if 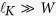, Eq. (1) can be approximated as 
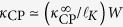
, i. e., *κ*_CP_ increases linearly with *W*, with a slope, 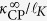, which decreases by increasing the ratio *m*_*B*_/*m*_*A*_. For instance, it is 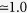 for *m*_*B*_/*m*_*A*_ = 2 and 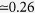 for *m*_*B*_/*m*_*A*_ = 4. For *m*_*B*_/*m*_*A*_ = 8, the slope is so small that *κ*_CP_ appears almost flat when plotted against *W*, for 20 ≤ *W* ≤ 40 (Fig. 2(c)).

The data shown in Fig. 2 demonstrate that *κ*_CP_ can be indeed lowered below the disordered alloy limit for *m*_*B*_/*m*_*A*_ = 2, and even below the glass limit for higher mass heterogeneities, *m*_*B*_/*m*_*A*_ = 4 and 8. These results are consistent with the experimental work of Ref. 18, and demonstrate that the interface formed between dissimilar materials effectively reduces *κ*_CP_. It is also worth noting that the thermal conductivity tensor is very strongly anisotropic in this case, with 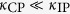.

**Figure 3:**
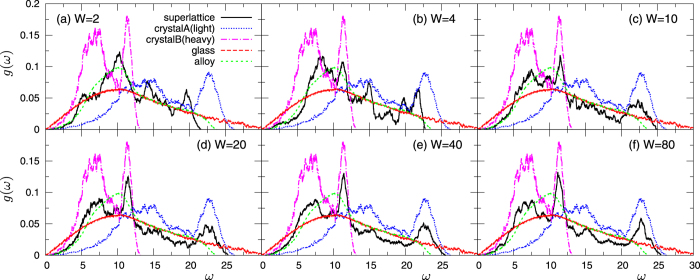
Vibrational density of states in superlattice *S1*, for a mass ratio *m*_*B*_/*m*_*A*_ = 4 with *m*_*A*_ = 0.4 and *m*_*B*_ = 1.6. In panels (**a**–**f**) we show the data corresponding to the repetitions period values *W* = 2,4,10,20,40,80. For comparison, we also plot *g*_*A*(*B*)_(*ω*) for the homogeneous bulk crystal composed by light (heavy) *m*_*A*(*B*)_ masses only, together with the data for the glass and the disordered alloy formed by the same constituent species.

The vibrational modes of the structure, i.e., the superlattice phonons, are key to understand the above behaviour of thermal conductivity. In Fig. 3 we show the vibrational density of states (vDOS), *g*(*ω*), for *m*_*B*_/*m*_*A*_ = 4 and *W* = 2 to 80. *g*_*A*_(*ω*) and *g*_*B*_(*ω*) of the bulk crystals of type *A* and *B* as well as the vDOS of the glass and of the disordered alloy are also shown for comparison. Note that 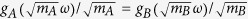. At small *W* = 2, *g*(*ω*) of the superlattice roughly follows that of the disordered alloy, implying that the vibrational states in the two layers are strongly mixed. In this situation, phonons are able to propagate in both the cross- and in-plane directions. On the other hand, as *W* increases, *g*(*ω*) generates features increasingly similar to those identifying *g*_*A*_(*ω*) and *g*_*B*_(*ω*), separately. In particular, in the low-*ω* region *g*(*ω*) follows *g*_*B*_(*ω*) (the heavy crystal *B*), whereas *g*_*A*_(*ω*) (the light crystal *A*) controls *g*(*ω*) in the high-*ω* region. This result indicates that different parts of the vibrational spectrum are active in the two layers, with high (low)-*ω* modes preferentially excited in the light (heavy) layer *A* (*B*). In this situation, phonon propagation is largely obstructed in the cross-plane direction, leading to the observed large reduction of *κ*_CP_. We remark that phonons propagate in the in-plane direction with small constraints, as shown by the large value of *κ*_IP_ close to 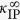. This implies that phonons, whose propagations are blocked in the cross-plane direction, are actually specularly reflected at the interface and confined in the in-plane direction.

**Figure 4:**
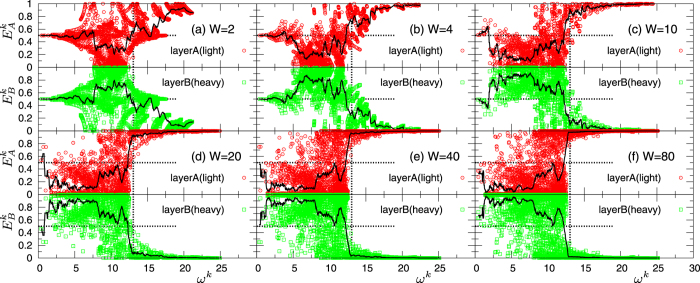
Vibrational amplitudes of normal modes in superlattice *S1*, for a mass ratio *m*_*B*_/*m*_*A*_ = 4 with *m*_*A*_ = 0.4 and *m*_*B*_ = 1.6. The vibrational amplitudes of the eigenvectors, 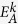 and 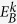, in layers *A* (light) and *B* (heavy) for all normal modes *k*, are plotted as functions of the corresponding eigenfrequency *ω*^*k*^. 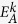 and 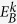 are defined in Eq. (6). The repetitions period values are *W* = 2,4,10,20,40,80 in panels (**a**–**f**). The solid line represents the average values 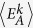 and 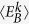 calculated in bins of the form *ω*^*k*^ ± *δω*^*k*^/2, with *δω*^*k*^ = 0.5. The horizontal dotted lines represent the threshold value 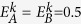, the vertical lines indicate 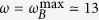, corresponding to the high frequency edge of *g*_*B*_(*ω*).

The separation of the vibrational states found in the *g*(*ω*) becomes more clear when considering the vibrational amplitudes associated with the eigenstates *k*. In Fig. 4 we show the vibrational amplitudes, 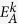 and 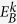 (Eq. (6)), in the two layers *A* and *B* for each mode *k*, together with the binned average values (solid lines). Based on the relations 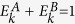 and 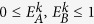, we can define a relative degree of excitation of particles in the two layers, by the threshold value 0.5: large excitations correspond to 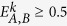, small excitations to 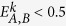. If 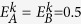, particle vibrations in both layers are of the same degree and correlated.

At small *W* = 2,4 we find, particularly in the low-*ω* region, a large fraction of vibrational states with 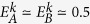. As *W* increases, in the high frequency region 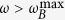, where 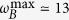 is the high-frequency boundary in *g*_*B*_(*ω*), only particles in the light layer *A* vibrate (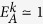), whereas those in the heavy layer *B* are almost immobile, as indicated by 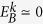. In this *ω*-region, phonon propagation in the cross-plane direction is therefore almost completely suppressed. On the other hand, for 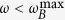, particles pertaining to the heavy layer *B* show large vibrational amplitudes (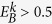), while vibrations in layer *A* tend to be small (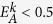). More in details, for *W* ≥ 20, we see that the averaged amplitudes are much larger in the *B* layer (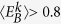) than in the *A* layer (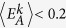) in the 2 < *ω* < 7.5 range. Contrary to the case of 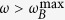, however, a significant number of modes are excited in both layers *A* and *B*, even with 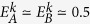. We therefore conclude that, for 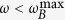, some phonons still propagate in the cross-plane direction, contributing to *κ*_CP_.

We note that our observation of the vibrational separation in both the vDOS and vibrational amplitudes is consistent with results reported previously^27^,^41^,^50^. Indeed, the simulation work of Ref. 27 reported a separation in the vDOS of the Si isotopic-superlattice (^28^Si-^42^Si superlattice). A recent simulation work^41^ focused on partial inverse participation ratios in a superlattice similar to the one considered here, reporting vibrational modes separation between layers. Ref. 50 attributed the reduction of thermal conductivity to a mechanism described as phonon localization, which we consider to be essentially the same phenomenon as the vibrational separation described here.

We believe that this concept of vibrational separation is a simple and accurate framework to rationalize the behaviour of thermal conductivity in superlattices. In particular, it provides a complete characterization of the minimum in the *W*-dependence of *κ*_CP_. Indeed, in the range *W* = 2 to 20 identifying the coherent regime, the vibrational separation hinders the coherent phonon propagation in the cross-plane direction, leading to the large reduction of *κ*_CP_. In contrast, in-plane phonon propagation is very mildly affected by the vibrational separation and, therefore, *κ*_IP_ keeps high values. Also, by considering 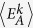 and 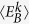 (solid lines), we conclude that the separation saturates to its maximum level at 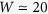. Upon further increase *W* > 20, although averaged values show no significant changes, we recognize an increasing fraction of modes with 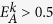 and 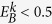 for 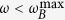 (panels (e) *W* = 40 and (f) *W* = 80 in Fig. 4). This observation indicates that the separation tendency for modes with 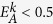 and 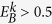 becomes weaker, i.e., the correlation of vibrational features in the two layers decreases, which corresponds exactly to the incoherent transport picture, and leads to the increase of *κ*_CP_. Although transport becomes completely incoherent only for values of *W* of the order of the Kapitza length (note that 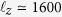 for *m*_*B*_/*m*_*A*_ = 4), this feature appears as soon as the vibrational separation is saturated, at the crossover point 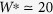. Thus, the saturation point of the vibrational separation identifies the minimum value of *κ*_CP_, which can be indeed below the glass limit.

### S2. Superlattice composed of intercalated ordered crystalline layers and mass disordered alloy layers

This system consists of three components, with masses *m*_*A*_ = 1 in the crystalline layer *A*, and *m*_*B*1_ and *m*_*B*2_ in the disordered alloy layer *B*. Note that also in this case, on the basis of the same arguments discussed above for *S1*, the average mass in layer *B* and that of the entire system are fixed as 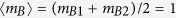 and 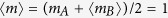, respectively, in order to eliminate the trivial contribution associated with different average values in the different cases.

**Figure 5:**
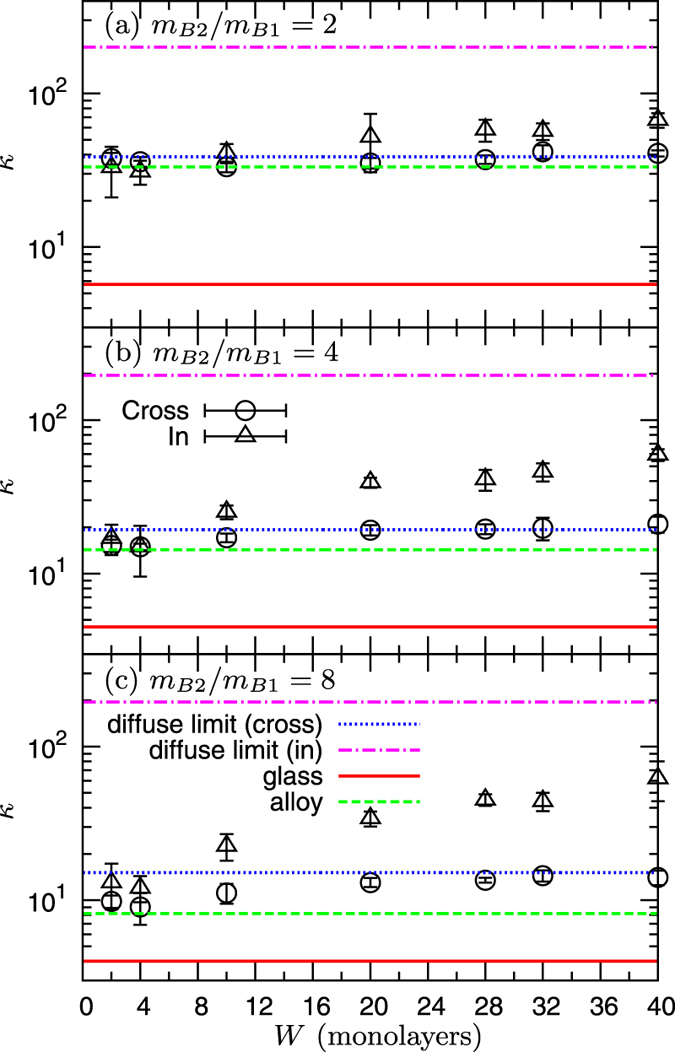
Thermal conductivity in superlattice *S2* composed of ordered crystalline layers intercalated with mass disordered alloy layers. The two components *κ*_CP_ and *κ*_IP_ are plotted as functions of *W*. The mass ratio of the disordered alloy layer *m*_*B*2_/*m*_*B*1_ is (**a**) 2, (**b**) 4, and (**c**) 8. The values 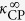 and 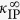 of the diffuse limits, together with those in the glass and the disordered alloy are indicated by lines.

**Figure 6:**
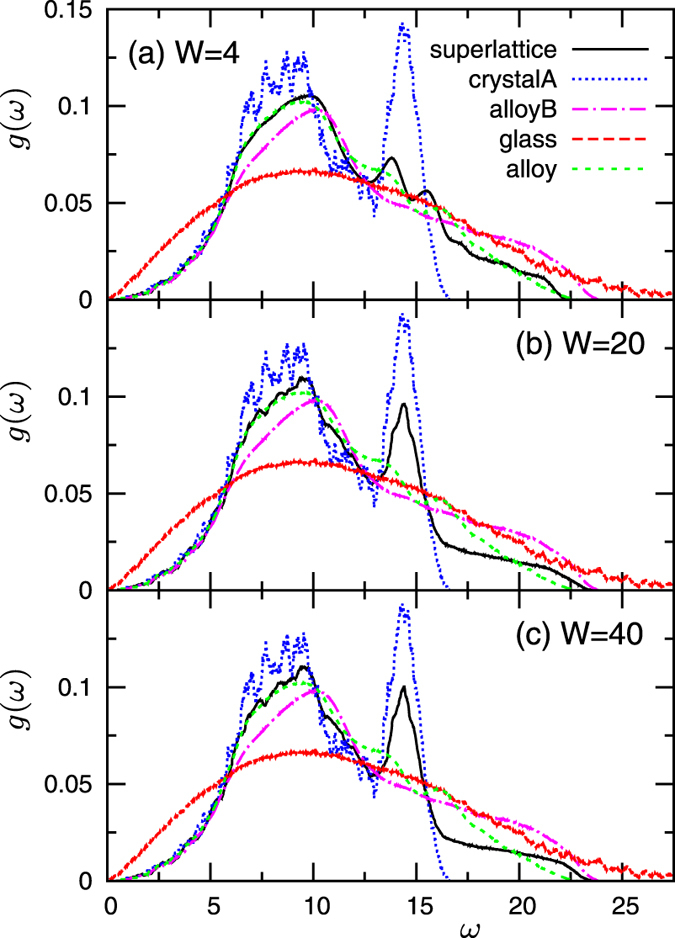
Vibrational density of states in superlattice *S2*. We show our results for the mass ratio of the disordered alloy layer *m*_*B*2_/*m*_*B*1_ = 4, with *m*_*B*1_ = 0.4 and *m*_*B*2_ = 1.6. The period *W* is 4, 20, and 40 for (**a**–**c**), respectively. For comparison, we plot *g*_*A*_(*ω*) of the bulk crystal formed by particles of mass *m*_*A*_ = 1 (crystal *A*), *g*_*B*_(*ω*) of the disordered alloy with masses *m*_*B*1_ = 0.4 and *m*_*B*2_ = 1.6 (alloy *B*), and the vDOS of the glass and the disordered alloy formed by the same constituent species.

In Fig. 5, we plot *κ*_CP_ and *κ*_IP_ for the mass ratios of the layer *B*, *m*_*B*2_/*m*_*B*1_ = 2, 4, and 8. At small *W* ≤ 4, the values of both *κ*_CP_ and *κ*_IP_ are very close to those of the disordered bulk alloy formed by the same particles. As *W* increases, *κ*_IP_ increases gradually toward 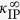. This increase is controlled by the development of in-plane phonon propagation in the ordered crystalline layer *A*. Indeed, the *g*(*ω*) of the superlattice, shown in Fig. 6, roughly follows that of the disordered bulk alloy at small *W* = 4, whereas at large *W* = 20,40 it is dominated by *g*_*A*_(*ω*). In particular, the longitudinal peak around 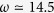 becomes clear, corresponding to that of the crystalline layer *A*.

The cross-plane value *κ*_CP_ also increases with *W*, but reaches the limit value 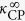 already at 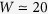 Since *κ*_*B*_ of the disordered alloy layer *B* is low (see Table 1), 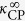 remains low, typically less than twice the disordered alloy value. As a result, the variation of *κ*_CP_ with *W* is small. This result indicates that scattering in the disordered alloy layer *B* dominates the thermal conduction in the cross-plane direction. Both experimental work^51^ on Si(crystal)-SiGe(disordered alloy) nanowires and numerical simulations^40^ have reported similar observations. We also note that the coherent nature of the superlattice phonons in the cross-plane direction, which we observed in the *S1* system, breaks down in *S2*. This is essentially equivalent to previous findings that disorder in interfacial roughness^37^,^38^,^39^, or interfacial species mixing^40^,^41^ destroy the coherent features of vibrational excitations present in the investigated superlattices. As a consequence of these features, in superlattices of type *S2* the variability of the cross-plane heat transport is strongly bounded, and the minimum limit of *κ*_CP_ just corresponds to the disordered alloy limit, i.e., *κ*_CP_ cannot be reduced below the glass limit.

Finally it is worth to mention that the thermal conductivity tensor becomes increasingly anisotropic at larger *W* due to the increase of *κ*_IP_, showing a behaviour different than that observed in *S1* where the anisotropy reaches the maximum at the crossover point 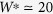.

### S3. Superlattice composed by identical crystalline layers separated by weakly interacting interfaces

**Figure 7:**
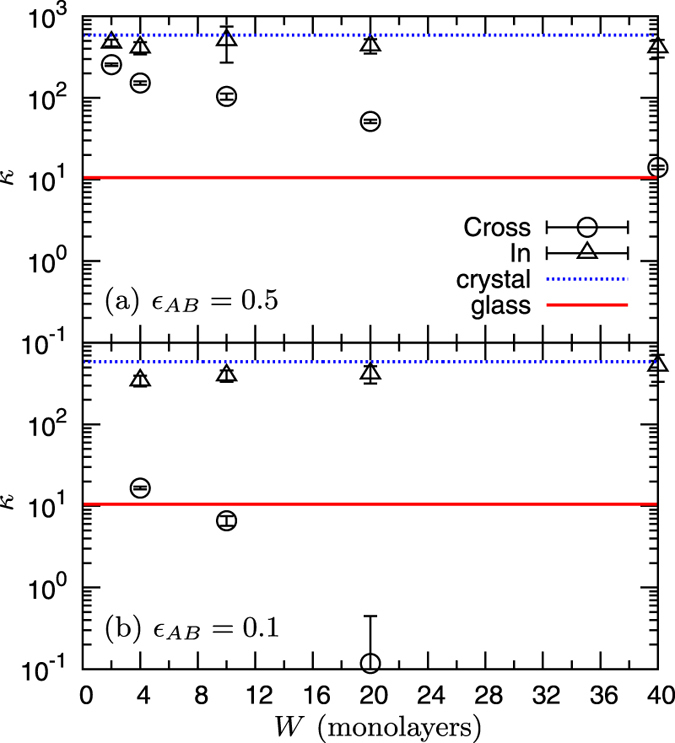
Thermal conductivity in superlattice *S3* composed of identical crystalline layers with weakened interface. The two components *κ*_CP_ and *κ*_IP_ are plotted as functions of *W*. The interface interaction *ε*_*AB*_ is 0.5 in (**a**) and 0.1 in (**b**). We also show, by the horizontal lines, the thermal conductivities of the corresponding one-component homogeneous bulk crystal and glass with unmodified interactions.

In Fig. 7 we show the *W*-dependences of *κ*_CP_ and *κ*_IP_ for the case where the energy scale associated with particles interactions across the interfaces (*ε*_*AB*_) are lowered compared to those intra-layers, with *ε*_*AB*_ = 0.5 and 0.1 in the two panels. In the figure, we also plot as lines the data for the corresponding one-component crystal and glass with unmodified interactions. Taking the one component system as reference is justified on the basis of earlier work^8^. There, it was shown that when a system of soft spheres is frozen in a disordered state, slightly modifying even a substantial fraction of the interactions does not have any appreciable influence on the vibrational properties, including thermal transport. As a consequence, in the present case we do not expect any relevant modification to *κ* originating from a limited fraction (scaling with the surface of the interfaces) of modified interactions in the glass sample.

The in-plane value *κ*_IP_ is almost independent of *W*, and is very close to the value pertaining to the crystal. In contrast, *κ*_CP_ decreases monotonically by increasing *W*, and especially in the weaker case *ε*_*AB*_ = 0.1, the observed reduction of *κ*_CP_ is dramatic. At *W* = 10, *κ*_CP_ equals the value obtained for the glassy sample, and it is almost two orders of magnitude lower than this value at *W* = 20. This extremely low *κ*_CP_ is consistent with previous experimental work^19^.

**Figure 8:**
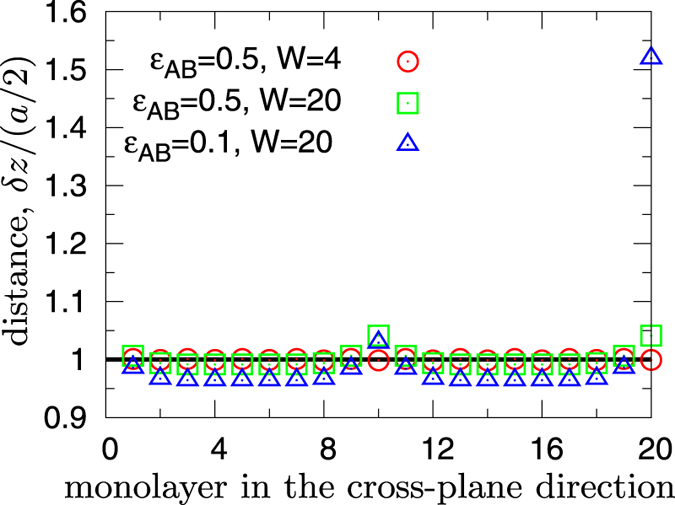
Distance between adjacent monolayers in superlattice *S3*. The average cross-plane distance *δz* between adjacent crystalline planes plotted for each monolayer, identified by the corresponding order index. We present the value of *δz* normalized to *a*/2, the horizontal line *δz*/(*a*/2) = 1 therefore indicates the value in the perfect crystalline lattice. The displacements observed in the cases *W* = 20 are discussed in the main text.

Some insight about the origin of this observation comes from the data shown in Fig. 8, where we display the average cross-plane distance *δz* between adjacent crystalline planes (monolayers), normalized to the value in the perfect lattice, *a*/2. For *ε*_*AB*_ = 0.5 and *W* = 4, the system keeps the perfect lattice structure, with *δz* ≡ *a*/2 for all monolayers. In contrast, as *ε*_*AB*_ decreases and for a large value *W* = 20, *δz* becomes substantially larger than *a*/2 at the interfaces, which therefore assumes a local density lower than the average. At the same time, slightly reduced *δz* are also observed for the other intra-monolayers, leading to an increase of the local density compared to the average. This heterogeneity hinders energy propagation across the interface and, as a result, phonons are specularly reflected and confined in the in-plane direction. We remark that in the cases with *W* = 20, the values of *δz* at the interfaces located at *W*/2 = 10 and *W* = 20 are different, with a large discrepancy for *ε*_*AB*_ = 0.1. We rationalize this behaviour by observing that, during the preparation stage of the sample, the applied selective weakening of the interactions destabilizes the global equilibrium of the superlattice, with a concentration of mechanical stress close to the interfaces. Lattice planes far from the boundaries easily recover mechanical equilibrium by coherently reducing their mutual distance. In contrast, particles in monolayers adjacent to the interfaces move both out-of-plane and in-plane, to optimize the local effective spring constants. The optimal solution found depends in general on the details of the local environment, explaining the observed discrepancy in *δz* at different interfaces.

**Figure 9:**
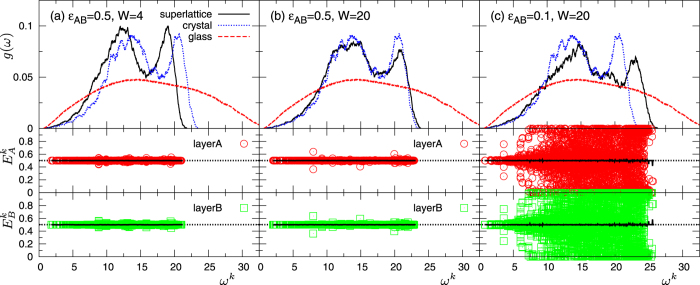
Vibrational density of states and vibrational amplitudes in superlattice *S3*. We report data corresponding to the indicated values of the the interfacial interaction energy *ε*_*AB*_ and the repetition period *W*: (**a**) *ε*_*AB*_ = 0.5, *W* = 4, (**b**) *ε*_*AB*_ = 0.5, *W* = 20, and (**c**) *ε*_*AB*_ = 0.1, *W* = 20. In panels at the top, we show the vDOS *g*(*ω*) for the superlattices of type *S3*, together with those of the corresponding one-component homogeneous crystal and glass with unmodified interactions. In panels at the bottom we show the vibrational amplitudes, 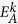 and 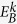, in layers *A* and *B*, plotted as functions of the eigenfrequency *ω*^*k*^. The solid line represents the average values 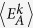 and 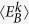, calculated in bins of the form *ω*^*k*^ ± *δω*^*k*^/2, with *δω*^*k*^ = 0.5. The horizontal dotted lines indicate 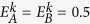.

The behaviour of *κ*_CP_ can be further elucidated by inspection of the main features of the vibrational spectrum. In Fig. 9 we plot the *g*(*ω*) of superlattice *S3*, together with the vibrational amplitudes 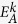 and 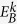. The transverse and longitudinal phonon branches are observed in *g*(*ω*) (top panels) for all cases, similar to the homogeneous bulk crystal. For *ε*_*AB*_ = 0.5, *W* = 4, *g*(*ω*) shows an excess of lower-*ω* modes compared to those present in the one-component crystal, simply due to the weakened interactions at the interfaces. As *ε*_*AB*_ decreases and *W* increases, *g*(*ω*) deforms, following the appearance of an increasing fraction of modes at increasing higher frequencies. This behaviour is certainly correlated to the observation made above (see Fig. 8) for the cases of *ε*_*AB*_ = 0.5, *W* = 20 and *ε*_*AB*_ = 0.1, *W* = 20, that the distance between monolayers far from the interfaces becomes smaller than *a*/2. The consequent larger mass density makes higher the frequency of phonon modes of given wavelength, leading to the shift of *g*(*ω*) towards higher frequencies.

We now focus on the vibrational amplitudes, 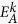 and 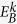 (Fig. 9, bottom panels). In the cases with *ε*_*AB*_ = 0.5 and *W* = 4 and 20, the particles in the two layers *A* and *B* show completely equivalent and correlated vibrations for the vast majority of the modes, as indicated by 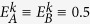. This result implies that phonons indeed propagate across the weakened interfaces in the cross-plane direction, but they are also partially reflected at the interface, causing the observed reduction of *κ*_CP_. The situation changes drastically in the case *ε*_*AB*_ = 0.1 and *W* = 20, where the ultra-low value of *κ*_CP_ can be reached. Except for the low-*ω* modes, 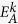 and 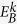 are symmetrically randomly distributed around the average values 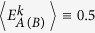, indicating that particles in layers *A* and *B* vibrate independently, in an uncorrelated manner. As a consequence, a very large fraction of vibrational modes do not cross at all the interfaces, but rather undergo a perfect specular reflection. In this situation, heat is not transferred between two adjacent layers *A* and *B*, leading to extremely low value of *κ*_CP_, while keeping a high *κ*_IP_.

We conclude by noticing that although specular reflection was also observed in the system *S1*, the physical mechanism behind this phenomenon is different in the two cases: vibrational separation causes reflection in the former, whereas weakened interactions across the interface, with the resulting augmented spacing between the layers, completely block cross-plane phonon propagation in the latter.

## Discussion

We have provided numerically, for the first time to our knowledge, a clear demonstration of very low thermal conductivities in superlattices, below the glassy limit of the corresponding amorphous structures. Blocking phonon propagation in ordered structures via interfaces design is the key principle. We have identified two possible strategies to achieve this goal: imposing a large mass heterogeneity in the intercalated layers (as in system *S1*) or degrading inter-layers interactions compared to those intra-layers (as in *S3*). We have found that in both cases phonons are specularly reflected at the interface and confined in the in-plane direction. This reduces the cross-plane thermal conductivity *κ*_CP_ below the corresponding glass limit, while keeping the in-plane contribution *κ*_IP_ close to the pure crystalline value.

More specifically, in the case of mass mismatch (*S1*), propagation of phonons with high frequencies (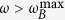) is almost completely suppressed, whereas a fraction of low-frequency phonons (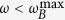) are still able to propagate across the interfaces, contributing to *κ*_CP_ (Fig. 4(d–f)). Also, the minimum in thermal conductivity as a function of the repetition period *W* (Fig. 2) corresponds to a maximum in the vibrational separation between the layers of type *A* and *B*. These therefore act as true filters in complementary regions of the vibrational spectrum, suppressing significantly phonons transport in the direction of the replication pattern. On the other hand, attenuated interactions across the interfaces (*S3*) are able to block phonons at a very wide range of frequencies (see Fig. 9(c)), which results into extremely low values of *κ*_CP_, even orders of magnitude lower than the corresponding glass limit (Fig. 7(b)). In this sense, directly modifying the interfaces seems to be the most effective strategy to obtain very low heat transfer. Note that this is a practically feasible route, since attenuated interfaces can be designed by exploiting materials with weak van der Waals forces among adjacent crystalline planes, as demonstrated in the case of WSe_2_ sheets in Ref. 19. Interfaces stiffness modification by controlling pressure^44^,^45^ or chemical bonding^46^ are additional possible routes to directly tune the strength of interfaces.

Our data also suggest that intercalating disordered alloy layers in ordered crystalline layers (*S2*) is not effective in lowering *κ*_CP_. Indeed, we have demonstrated that in this case disorder is not sufficient to block the propagation of vibrational excitations, even though it makes phonons lifetimes short. The intercalated disordered alloy layer dominates phonon transport in the entire superlattice, notwithstanding the presence of the crystalline layers. As a result, thermal conductivity is very similar to the one of the disordered alloy and is only marginally modified by modulation of the period *W* (see Fig. 5). Also, as suggested in previous works, disorder in the interfacial roughness^37^,^38^,^39^ or interfacial mixing^40^,^41^ seems to already dominate over phonon transport, and destroy the coherent nature of phonons.

In addition, as we understand from our analysis of vibrational amplitudes (Figs 4 and 9), it is much more problematic to block low-*ω* (long wavelength, *λ*) phonons propagation, than those with high-*ω* (short *λ*). This situation is similar to what has been observed in bulk glasses, where the long-*λ* acoustic waves are not scattered by the disorder and can propagate over long distances by carrying heat energy^49^,^52^. Therefore, blocking or efficiently scattering the long-*λ* phonons is also a key factor to achieve very low thermal conductivities, as was pointed out in Ref. 53. A possibility to realize this task is embedding in the targeted material objects featuring larger typical sizes, including nano-particles^54^,^55^ or nano(quantum)-dots^20^,^56^. Based on this strategy, very low thermal conductivity was achieved experimentally in a Si-Ge quantum-dot superlattice^20^, even below the amorphous Si value. The additional possibility of introducing large size defects by the porous structuring of materials has also been explored in a recent numerical work^57^. Here, values of thermal conductivity 10^4^ times smaller than that of bulk Si were reached in Si phononic crystals with spherical pores.

An other important point must be underlined. In the present work we have investigated the different systems at low temperature, to both focus on plainly structural effects and keep contact with the well controlled harmonic limit. Anharmonicities, however, are expected to play a crucial role at temperatures higher than the Debye temperature, *T*_*D*_, which are those relevant for technological applications. This is a crucial aspect to be explored in extended future work.

In conclusion, we note that the three superlattice structures studied in the present work show totally different *W*-dependences of cross and in-plane thermal conductivities. Our results therefore not only contribute to a deeper understanding of the physical mechanisms behind very-low thermal conductivity, they also provide insight for developing new design concepts for materials with controlled heat conduction behaviour.

## Methods

### System description

In this Section we provide details on the numerical models we have used for the superlattices. The corresponding amorphous structures (glasses) and disordered alloys with exactly the same composition were also prepared, for the sake of comparison with superlattice phases. We have considered in all cases a 3-dimensional cubic box, of volume *V* = *L*^3^ (*L* being the linear box size), with periodic boundary conditions in all directions. In the superlattice and disordered alloy cases, particles were distributed on the FCC lattice sites. In the glass phases, they were frozen in topologically random positions following a rapid quench from the normal liquid phase below the glass transition temperature *T*_*g*_, avoiding crystallization (see, for instance, Ref. 49 for details on the preparation of glasses). Particles, *i* and *j*, interact via soft-sphere (SS) or Lennard-Jones (LJ) potentials:


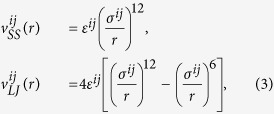


where *r* is the distance between those two particles, and *σ*^*ij*^ and *ε*^*ij*^ are the interparticle diameter and interaction energy scale, respectively. The potential is cut-off and shifted at *r*_*c*_ = 2.5*σ*^*ij*^. Particle *i* has mass *m*^*i*^, and we have used *σ*, *ε*/*k*_*B*_ (*k*_*B*_ is the Boltzmann constant), and *m* as units of length, temperature, and mass. As a reference, for Argon *σ* = 3.4 *Å*, *ε*/*k*_*B*_ = 120 K, and *m* = 39.96 a.m.u. We considered the number density 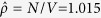, corresponding to a lattice constant 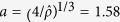.

We prepared three superlattices, composed of intercalated FCC lattice layers, *A* and *B*, both of thickness *W*/2, as schematically illustrated in Fig. 1. The first superlattice (*S1*) consists of two crystalline layers formed by point particles with different masses, *m*_*A*_ and *m*_*B*_. We have considered mass ratios *m*_*B*_/*m*_*A*_ > 1, while keeping a constant average mass (*m*_*A*_ + *m*_*B*_)/2 = 1. As an example, the case *m*_*B*_/*m*_*A*_ = 4 corresponds to *m*_*A*_ = 0.4 and *m*_*B*_ = 1.6. We have dubbed *A* and *B* as the light and heavy layers, respectively. Note that a mass ratio of *m*_*B*_/*m*_*A*_ = 2.5 corresponds to the case of the realistic Si-Ge superlattice. Except for the above mass difference in the different layers, all particles are characterized by the same properties. In particular, they interact via the SS potential 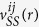, with *σ*^*ij*^ = *ε*^*ij*^ = 1.

The second superlattice (*S2*) is composed of an ordered crystalline layer *A* intercalated to a disordered alloy layer *B*. *m*_*A*_ = 1 in *A*, whereas in *B* half of the particles have mass *m*_*B*1_, *m*_*B*2_ the others, and are randomly distributed on the lattice sites. Again, *m*_*B*1_ and *m*_*B*2_ are determined by the mass ratio *m*_*B*2_/*m*_*B*1_> 1, keeping a constant average value (*m*_*B*1_ + *m*_*B*2_)/2 = 1. All particles in both layers interact via the SS potential 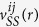 with *σ*^*ij*^ = *ε*^*ij*^ = 1.

The third superlattice (*S3*) is composed of identical crystalline layers *A* and *B*, but the interactions among particles in different layers (i.e., across the interfaces) are modified (weakened) compared to those intra-layers. All particles have mass *m*_*A*_ = *m*_*B*_ = 1, and interact via the LJ potential 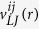, with *σ*^*ij*^ = *ε*^*ij*^ = 1. The energy scale of interactions between particles pertaining to different layers are, however, reduced to *ε*^*ij*^ = *ε*_*AB*_ < 1.

### MD simulation and the Green-Kubo method for the calculation of thermal conductivity

In the present study, all simulations have been realized by using the large-scale, massively parallel molecular dynamics simulation tool LAMMPS^58^. The systems were first equilibrated at relatively low temperature *T* = 10^−2^ by MD simulation in the *NVT*-ensemble. This choice was dictated by the need to reduce anharmonic effects, in order to primarily focus on the contribution of the structural features of the superlattices on thermal conductivity. We must note that our approach is classical, and does not take into account the quantum mechanisms active in the low-*T* regime^9^. These effects have important implications, increasing the contribution to the thermal conductivity coming from low-*ω* vibrational excitations. At present, however, it is not obvious and still under debate how to effectively include quantum effects into a classical system^59^,^60^, and we have therefore chosen to stay within a fully classical approach.

Following the equilibration stage, we performed the production runs in the *NVE*-ensemble. The Green-Kubo formulation^47^,^48^ was next applied to calculate the thermal conductivities, in the cross-plane and in-plane directions, respectively:


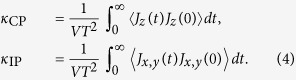


Here, *J*_*x*,*y*_ and *J*_*z*_ are the heat currents in the in-plane (*x*,*y*) and cross-plane (*z*) directions, and 〈〉 denotes the ensemble average. Landry *et al.*^48^ have carefully confirmed the validity of the Green-Kubo method for the calculation of superlattices thermal conductivity, by comparison with the direct method based on non-equilibrium simulation. In the bulk glasses and disordered alloys, 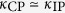, i.e., heat conduction is isotropic, whereas in the superlattices, they are expected to assume different values^16^,^17^.

More in details, the equations of motions were integrated numerically with a time step, *δt* = 5 × 10^−3^, for a total run-time *t*_run_ = *N*_run_*δt* = 10^5^ (*N*_run_ = 2 × 10^7^ steps) for *S1* and *t*_run_ = 10^4^ (*N*_run_ = 2 × 10^6^ steps) for both *S2* and *S3*. The systems snapshots extracted from the trajectory have been used to calculate the correlation functions, 
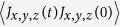
 of Eq. (4), which have been subsequently integrated numerically over a finite time window Δ*t*. We have observed a clear convergence of the integrals for *κ*_CP,IP_, to the limits of Eq. (4) for Δ*t* = 10^4^ for *S1*, and Δ*t* = 10^3^ for both *S2* and *S3*. For each considered superlattice, and all repetition periods *W*, we have performed 10 independent calculations starting from different initial system configurations. These has allowed us to obtain 10 independent sample values for *κ*_CP_ and 2 × 10 = 20 (we have considered both the *x* and *y* components) for *κ*_IP_. These values were used to calculate averages and sample-to-sample fluctuations (standard deviations), which are shown as error bars in Figs. 2,5 and 7. More details on these calculations are reported in the Supplementary Materials.

### Analysis of finite system size effect on thermal conductivity

In the Green-Kubo calculations of thermal conductivities, one must be attentive to finite system size effects^47^,^48^. Indeed, long-wavelength phonons with *λ* > *L* are excluded from the simulation box due to the finite value *L* of the box size, which imposes important size effects on the numerical determination of *κ*. The box size therefore needs to be large enough to include a vibrational spectrum sufficient to establish an accurate description of anharmonic coupling (scattering) processes^47^. We note that the considered *T* = 10^−2^ is low enough to substantially reduce anharmonic effects, but anharmonic couplings are still present. We can take care of finite size effects by increasing *L* to values where *κ*_CP_ and *κ*_IP_ become *L*-independent. For the glass and disordered alloy thermal conductivities, we have confirmed that a system size *L* = 10*a* (*N* = 4,000) is sufficiently large to obtain correct values of 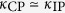, without any size effect^8^.

In the superlattice cases, the appropriate *L* depends on the considered structure and the periodic repetition length *W*^48^. More in details, we paid particular attention to the number *P* of repetitions, defined from *L* = *PW*, necessary to produce sufficient anharmonic couplings of phonons in the cross-plane direction. We have therefore investigated the presence of finite size effects by analyzing different systems with sizes ranging from *L* = 10*a* (20 monolayers, *N* = 4,000) to 24*a* (48 monolayers, *N* = 55,296). We provide details of our analysis of the finite-size effects in the Supplementary Materials. In Figs. 2,5 and 7, we plot the values obtained by using the largest systems (the exact system size depends on both the superlattice type and *W*), which show the smallest finite-size effects.

For the *S1* superlattice, we confirmed that the required number *P* of repetitions becomes larger for smaller *W*^48^: one period (*L* = *W*) only is adequate for *W* ≥ 20, whereas four periods or more (*L* ≥ 4*W*) are required for *W* ≤ 8 (see the Supplementary Materials). We have therefore employed four pattern repetitions (*L* = 4*W*) for 10 ≤ *W* ≤ 12 and two (*L* = 2*W*) for 14 ≤ *W* ≤ 18. This behaviour is simple to rationalize by inspecting the data in Fig. 2, where the crossover between incoherent and coherent phonon transport occurs around 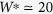. In the coherent regime *W* < 20, the wave character of the phonons becomes important, and therefore a larger number of repetitions is necessary to produce the coherent wave interference processes correctly. In contrast, smaller values of *P* are needed (even *P* = 1) in the incoherent regime *W* > 20, where the incoherent particle nature of the phonons appears.

For the *S2* and *S3* superlattices the system size effects issue is much less pronounced than in the *S1* case. We have confirmed that *P* = 1 or 2 (*L* = *W* or 2*W*) are sufficient for *W* ≥ 20, while two or more repetitions (*L* ≥ 2*W*) are appropriate for *W* < 20, for both *S2* and *S3* (see the Supplementary Materials). We can understand this behaviour by noticing that phonon transport is mainly determined by the scattering processes in the disordered alloy layer in *S2*, and the blocking at the weak interface for *S3*. In both cases the missing long wavelength phonons, with *λ* > *L*, play very little role in phonon transport and finite system size effects are consequently negligible.

### Normal modes analysis

We have characterized the superlattice vibrational states (superlattice phonons) by performing a standard normal-mode analysis^8^,^49^. We have diagonalized the dynamical (Hessian) matrix calculated at local minima of the potential energy landscape, and obtained eigenvalues *λ*^*k*^ and eigenvectors (polarization vectors) 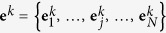. Here, *j* is the particle index, and *k* = 1, 2,…, 3*N* − 3 is the eigenmode number, where we have disregarded the three vanishing Goldstone modes. The eigenvectors are normalized such that 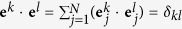, where *δ*_*kl*_ is the Kronecker delta function. The eigenfrequencies are next calculated as 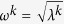, and the associated probability distribution (normalized histogram) directly provides the vDOS:


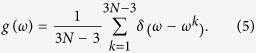


In addition, from the eigenvector ***e***^*k*^ we have defined the vibrational amplitudes of mode *k* for layers *A* and *B*:


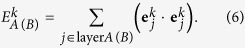


Note that 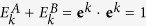 for each *k* and, therefore, 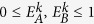. Based on the values of 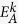 and 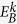, one can determine in which layer particles are more displaced (excited) according to the associated eigenvector ***e***^*k*^. In particular, if 
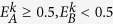
 (
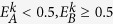
), particles in layer *A* (*B*) contribute more to mode *k* than those in layer *B* (*A*). In the case 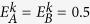, particles in both layers contribute equivalently, and in a correlated manner. Note that the normal mode analysis provides us with the system vibrational states in the harmonic limit *T* → 0 which, we believe, is an appropriate approximation for our case *T* = 10^−2^, where anharmonicities are weak.

## Additional Information

**How to cite this article**: Mizuno, H. *et al.* Beating the amorphous limit in thermal conductivity by superlattices design. *Sci. Rep.* **5**, 14116; doi: 10.1038/srep14116 (2015).

## Supplementary Material

Supplementary Information

## Figures and Tables

**Table 1:**
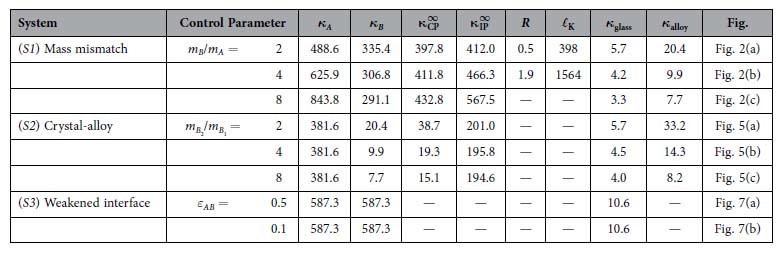
Details of the three superlattice systems investigated in this work.

They are based on the FCC-crystal lattice structure and are composed of: (*S1*) two intercalated crystalline layers (*A* and *B*) formed by point particles with different masses *m*_*A*_ and *m*_*B*_; (*S2*) ordered crystalline layers intercalated to mass-disordered alloy layers; and (*S3*) identical crystalline layers with modified (weakened compared to those intra-layers) interactions across the interfaces. The control parameters are the mass ratio *m*_*B*_/*m*_*A*_ in *S1*, the mass ratio *m*_*B*2_/*m*_*B*1_ of the disordered alloy layer in *S2*, and the energy scale *ε*_*AB*_ of the interactions across the interfaces in *S3*. Number density and temperature were fixed to the values 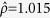 (corresponding to a lattice constant *a* = 1.58) and *T *= 10^−2^, respectively. The quantities presented in the table are defined in the main text. In the last column we refer to the figure containing the data relative to the indicated system. Additional details about the investigated superlattices and parameters used are given in the **Methods** section.
