# Supplementary material for: Beating the amorphous limit in thermal conductivity by superlattices design
Source: Sci Rep. 2015 Sep 16;5:14116. doi: 10.1038/srep14116 (PMC4571656; doi:10.1038/srep14116)
Supplement: Supplementary Information [file srep14116-s1.pdf]

Supplementary information for:

Beating the amorphous limit in thermal conductivity by superlattices design

by Hideyuki Mizuno, Stefano Mossa, and Jean-Louis Barrat

Email: stefano.mossa@cea.fr

Calculation of thermal conductivity by the Green Kubo method

We have performed the MD production runs in the NVE-ensemble, with a time step, $\delta t = 5 \times 10^{-3}$. The total production run time was $t_{\text{run}} = 10^5$ ($N_{\text{run}} = 2 \times 10^7$ steps) for the *S1* superlattice, and $t_{\text{run}} = 10^4$ ($N_{\text{run}} = 2 \times 10^6$ steps) for *S2* and *S3*. The obtained MD trajectory was subsequently used to calculate the auto-correlation function of the heat flux, $\langle J_\alpha(t) J_\alpha(0) \rangle$ [1]. Here, $J_\alpha(t)$ with $\alpha = x, y, z$ are the heat fluxes in x, y (in-plane, IP) and z (cross-plane, CP) directions, respectively, while $\langle\rangle$ denotes the ensemble average over the initial times $t = 0$. We next integrated the correlation functions over time, t, to obtain the time-dependent thermal conductivities [1],

$$\kappa_\alpha(t) = \frac{1}{VT^2} \int_0^t d\tau \langle J_\alpha(\tau) J_\alpha(0) \rangle. \tag{1}$$

Finally the thermal conductivities were determined by the "converged" values of $\kappa_\alpha(t)$ as,

$$\kappa_\alpha = \lim_{t \to \infty} \kappa_\alpha(t), \tag{2}$$

which provide the cross-plane, $\kappa_{\text{CP}} = \kappa_z$, and the in-plane, $\kappa_{\text{IP}} = \kappa_x,\ \kappa_y$, thermal conductivities.

In SFig. 1 we show the time-dependent thermal conductivities, $\kappa_x(t)$, $\kappa_y(t)$, and $\kappa_z(t)$, for the three studied superlattices. We note that in some cases (especially for *S1* in SFig. 1(c)), strong oscillations are present, and the convergence is not clear in the raw data, as also reported by a previous work on superlattices [2]. In order to overcome this difficulty, we have followed the scheme described in Ref. [1] and (roll-)averaged the data on overlapping time windows of appropriate widths (We considered, for instance, windows of 500 and 2000 MD steps for the data of SFig. 1(b) and (c), respectively).

For each superlattice and repetition period, W, we have performed 10 different calculations, starting from independent initial configurations. We therefore generated an ensemble of 10 independent values for each κ_α component, that we used for calculating both average values and error bars (standard deviations) for κ_{CP} and κ_{IP} shown in the figures of the articles.

Finite system size effect on thermal conductivity

In order to quantify finite system size effects on the reported thermal conductivity data, we have considered different systems, with sizes ranging from $L = 10a$ (20 monolayers and $N = 4,000$ particles) to $L = 24a$ (48 monolayers and $N = 55,296$). Following the procedure detailed above, we calculated data of κ_{CP} and κ_{IP} for all cases, which we plot in SFig. 2 as a function of the system sizes, L.

Note that particular attention must be devoted to the number of layers repetitions in the cross-plane direction, $P = L/W$ ($L = PW$) [2]. Indeed, P must be large enough for the simulation box to accommodate a sufficient fraction of relevant vibrational modes, particularly those propagating in the cross-plane direction.

Also, as it has been reported in Ref. [2], the appropriate P depends on the periodic repetition length, W: larger P values are necessary for smaller W. In SFig. 3 we plot the same data for $\kappa_{\rm CP}$ and $\kappa_{\rm IP}$ of SFig. 2, as a function of P. The two figures allow us to quantify the finite-size effects on our calculations, that we summarize as follows:

(i) For the in-plane thermal conductivity, $\kappa_{\rm IP}$, we do not recognize any noticeable dependences on L or P, for all the three superlattices. As a consequence, no finite-size effects seem to affect the values of $\kappa_{\rm IP}$.

(ii) The cross-plane values, $\kappa_{\rm CP}$, for *S2* and *S3*, also do not show any significant dependences on L or P. No serious size effects are evident also in this case, and we conclude that for *S2* and *S3*, $P = 1$ or 2 ($L = W$ or $2W$) are sufficient for $W \geq 20$, while two or more repetitions ($L \geq 2W$) are appropriate for $W < 20$.

(iii) In the $\kappa_{\rm CP}$ case for *S1*, no noticeable size-dependences are recognized for $W = 2,\ 4$ and $W = 20,\ 24$. In contrast, we have found a non-negligible effect for $W = 8,\ 12$. For $W = 8$, we have observed that finite-size effects are completely removed by choosing $L = 16a$ and $P = 4$. Therefore, we conclude that one period ($L = W$) only is already adequate for $W \geq 20$, whereas four periods or more ($L \geq 4W$) are required for $W \leq 8$. For the cases $10 \leq W \leq 18$, we employed four pattern repetitions ($L = 4W$) for $10 \leq W \leq 12$ and two ($L = 2W$) for $14 \leq W \leq 18$.

References

[1] McGaughey, A. J. H. & Kaviany, M. Phonon transport in molecular dynamics simulations: formulation and thermal conductivity prediction. *Advances in Heat Transfer* **39**, 169–255 (2006).

[2] Landry, E. S., Hussein, M. I. & McGaughey, A. J. H. Complex superlattice unit cell designs for reduced thermal conductivity. *Phys. Rev. B* **77**, 184302 (2008).

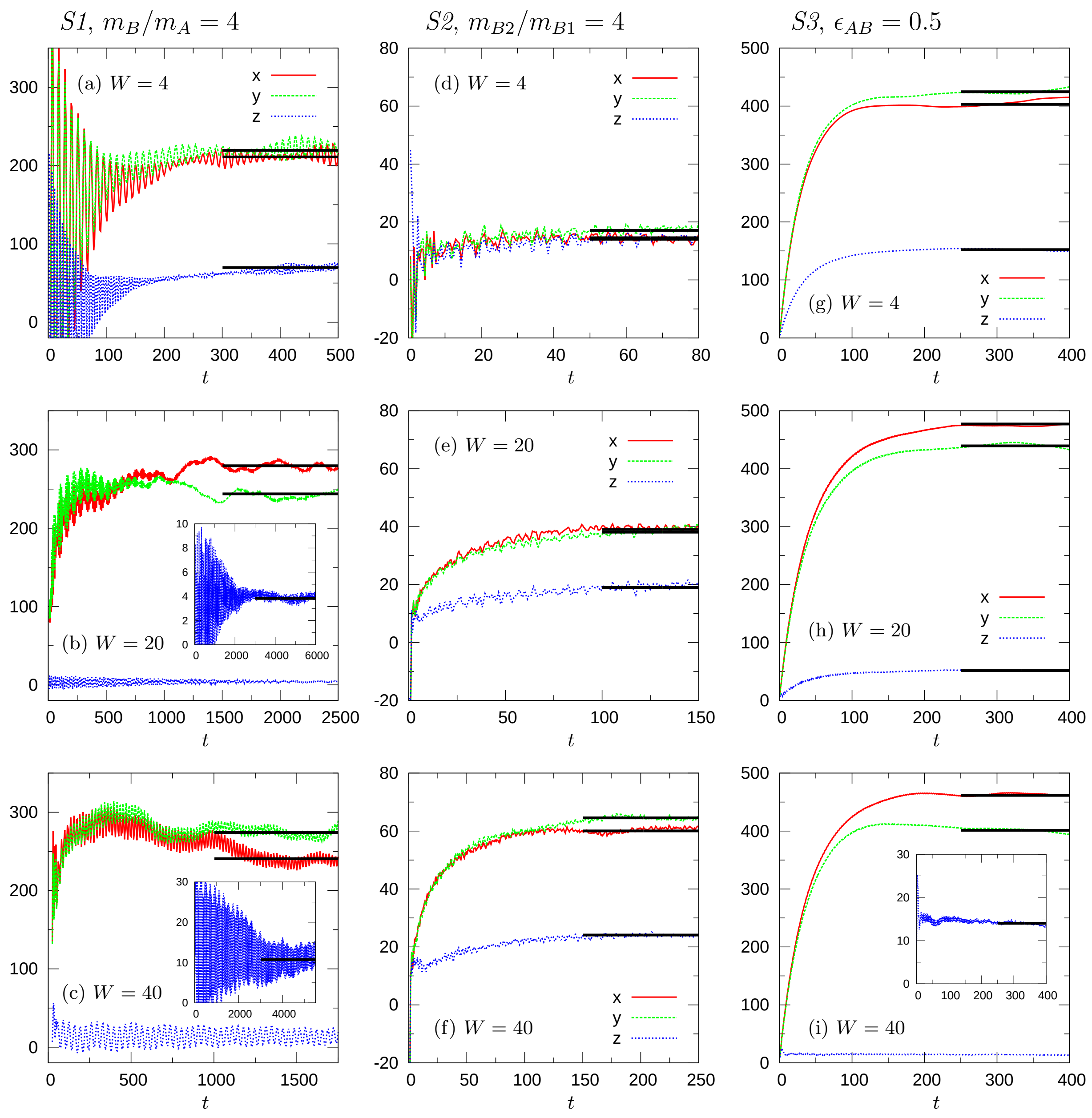

Supplementary Figure 1: The time-dependent thermal conductivities, $\kappa_x(t)$, $\kappa_y(t)$, and $\kappa_z(t)$, are plotted versus time, t, for the repetition periods $W = 4$ (top panels), $W = 20$ (middle panels), and $W = 40$ (bottom panels). The left ((a)-(c)), middle ((d)-(f)), and right ((g)-(i)) plots are for the superlattices *S1* ($m_B/m_A = 4$), *S2* ($m_{B2}/m_{B1} = 4$), and *S3* ($\epsilon_{AB} = 0.5$), respectively. In (b) and (c), the presented data are obtained by averaging the raw data on overlapping time windows, as discussed in the text. The horizontal thick lines indicate the converged final value for each case. The insets in panels (b), (c), and (i) are close-ups for the component $\kappa_z(t)$.

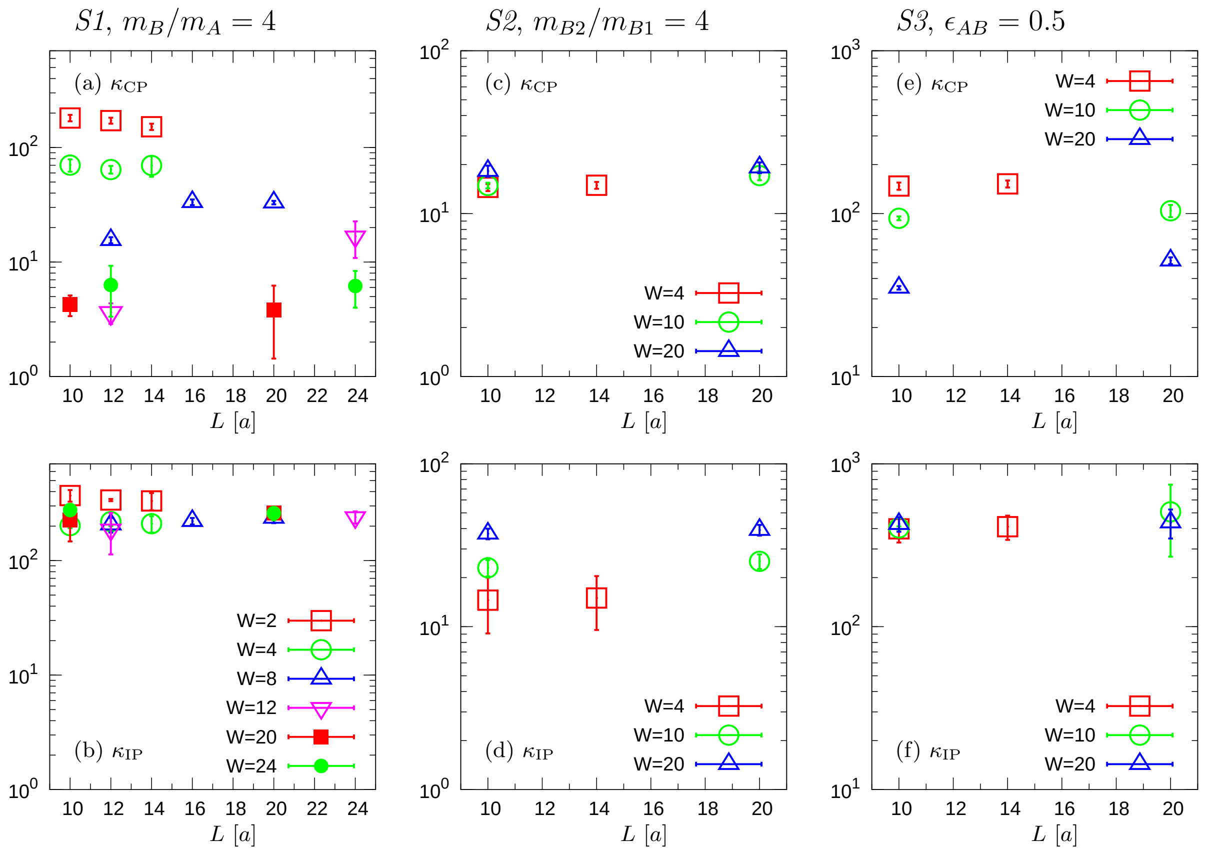

Supplementary Figure 2: The values of the thermal conductivities, $\kappa_{\rm CP}$ (top panels) and $\kappa_{\rm IP}$ (bottom panels), are plotted versus the system size L [a], for the indicated repetition periods W. The left ((a),(b)), middle ((c),(d)), and right ((e),(f)) panels are for the superlattices *S1* ($m_B/m_A = 4$), *S2* ($m_{B2}/m_{B1} = 4$), and *S3* ($\epsilon_{AB} = 0.5$), respectively. The error bars have been estimated as detailed in the text.

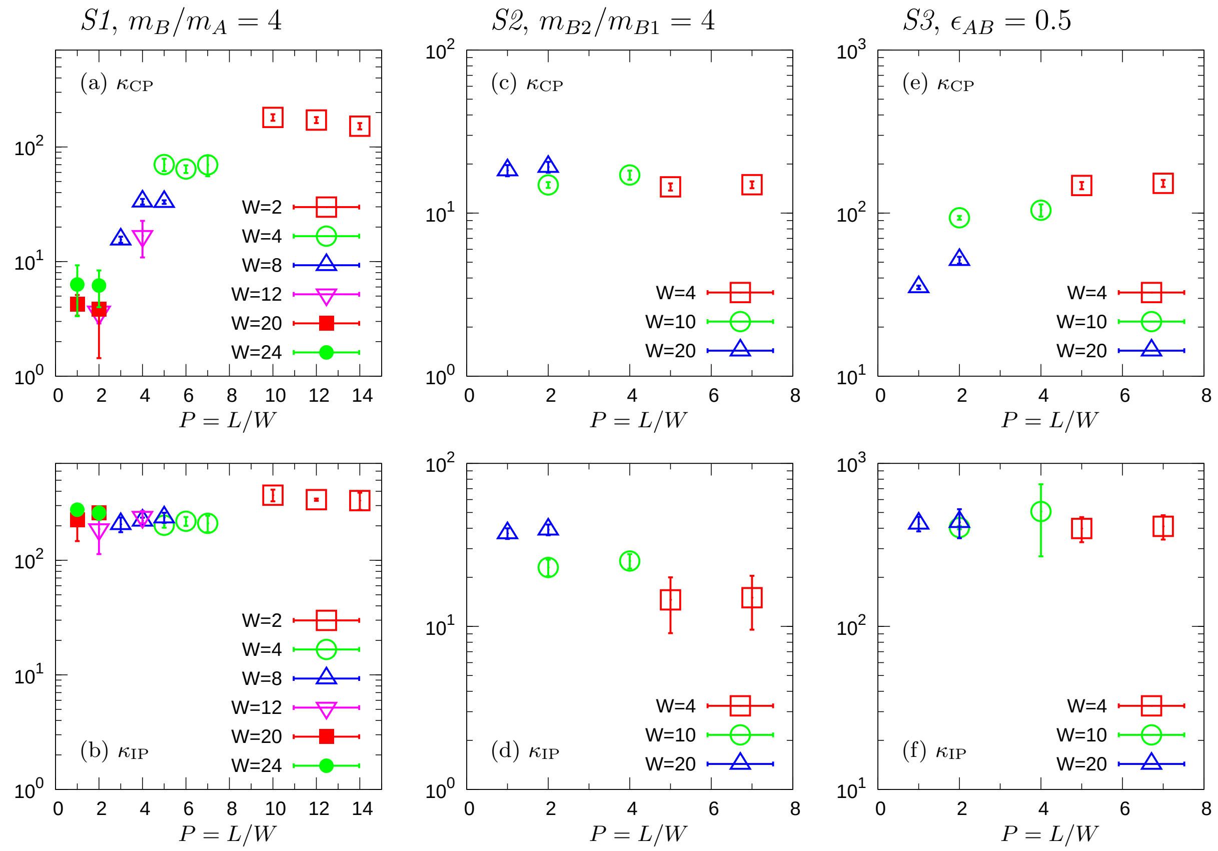

Supplementary Figure 3: The same data of SFig. 2 plotted versus the number of layers repetitions, $P = L/W$.
